# Transient ischemic attacks in patients with active and occult cancer

**DOI:** 10.3389/fneur.2023.1268131

**Published:** 2023-09-28

**Authors:** Morin Beyeler, Pasquale Castigliego, Joel Baumann, Victor Ziegler, Moritz Kielkopf, Madlaine Mueller, Stefan A. Bauer-Gambelli, Adnan Mujanovic, Thomas Raphael Meinel, Thomas Horvath, Urs Fischer, Johannes Kaesmacher, Mirjam R. Heldner, David Seiffge, Marcel Arnold, Thomas Pabst, Martin D. Berger, Babak B. Navi, Simon Jung, Philipp Bücke

**Affiliations:** ^1^Department of Neurology, Inselspital, Bern University Hospital, University of Bern, Bern, Switzerland; ^2^Graduate School for Health Sciences, University of Bern, Bern, Switzerland; ^3^Institute for Diagnostic and Interventional Neuroradiology, Inselspital, Bern University Hospital, University of Bern, Bern, Switzerland; ^4^Neurology Department, University Hospital of Basel, University of Basel, Basel, Switzerland; ^5^Department of Medical Oncology, Inselspital, Bern University Hospital, and University of Bern, Bern, Switzerland; ^6^Clinical and Translational Neuroscience Unit, Feil Family Brain and Mind Research Institute and Department of Neurology, Weill Cornell Medicine, New York, NY, United States; ^7^Department of Neurology, Memorial Sloan Kettering Cancer Center, New York, NY, United States

**Keywords:** transient ischemic attack, malignancy, biomarkers, cerebro-vascular disorders, D-dimer

## Abstract

**Background and aim:**

Paraneoplastic coagulopathy can present as stroke and is associated with specific biomarker changes. Identifying paraneoplastic coagulopathy can help guide secondary prevention in stroke patients, and early cancer detection might improve outcomes. However, unlike ischemic stroke, it remains unclear whether paraneoplastic coagulopathy is associated with transient ischemic attacks (TIA). This study assessed the presence of cancer-related biomarkers in TIA patients and evaluated long-term mortality rates in patients with and without active cancer.

**Methods:**

Active cancer was retrospectively identified in consecutive TIA patients treated at a comprehensive stroke center between 2015 and 2019. An association between the presence of cancer and cancer-related biomarkers was assessed using multivariable logistic regression. Long-term mortality after TIA was analyzed using multivariable Cox regression.

**Results:**

Among 1436 TIA patients, 72 had active cancer (5%), of which 17 were occult (1.2%). Cancer-related TIA was associated with male gender (adjusted odds ratio [aOR] 2.29, 95% CI 1.12–4.68), history of smoking (aOR 2.77, 95% CI 1.34–5.7), elevated D-dimer (aOR 1.77, 95% CI 1.26–2.49), lactate dehydrogenase (aOR 1.003, 95% CI 1.00–1.005), lower leukocyte count (aOR 1.20, 95% CI 1.04–1.38), and lower hemoglobin (aOR 1.02, 95% CI 1.00–1.04). Long-term mortality was associated with both active cancer (adjusted hazard ratios [aHR] 2.47, 95% CI 1.58–3.88) and occult cancer (aHR 3.08, 95% CI 1.30–7.32).

**Conclusion:**

Cancer-related TIA is not uncommon. Biomarkers known to be associated with cancer-related stroke also seem to be present in TIA patients. Early identification would enable targeted treatment strategies and could improve outcomes in this patient population.

## Introduction

Interest in cancer-related ischemic stroke has grown in recent years (1, 2). Pathophysiologically, it is predominantly attributed to paraneoplastic coagulopathy, which is defined as the expression of pro-coagulant factors generated by active cancer (2). The following pathways have been implicated: tissue factor, inflammatory cytokines, fibrinolysis inhibitors, extracellular vesicles, and extracellular neutrophil traps (2). In addition to intravascular coagulopathy, other factors such as paradox embolism, non-bacterial endocarditis as well as an increase in the presence of traditional stroke risk factors also seem to contribute (1). Recent studies suggested several blood and imaging biomarkers to be associated with underlying cancer in stroke patients: elevated levels of D-dimer, fibrinogen, C-reactive protein (CRP), and lactate dehydrogenase (LDH); low hemoglobin levels; and multi-territory ischemic infarction on brain imaging (3–5). The optimal stroke secondary prevention in cancer-related stroke is controversial. Some observational studies suggest that patients with cancer-related stroke may benefit from early anticoagulation (direct oral anticoagulants or low molecular weight heparin), but major guidelines advocate for randomized trials of anticoagulant vs. antiplatelet therapy (1, 6–8). However, it remains unclear whether the abovementioned paraneoplastic mechanisms and biomarkers are also associated with the occurrence of transient ischemic attack (TIA). Clinically speaking, there is little rationale to distinguish between TIA and ischemic stroke patients in terms of etiology, diagnostic evaluation, and secondary prevention (6). However, the identification of an underlying paraneoplastic coagulopathy in TIA could help guide secondary prevention in this population. TIA-like ischemic stroke may be the first manifestation of an unknown cancer, termed “occult cancer.” (3, 9). As underlying (known or occult) cancer is known to be associated with an increased rate of stroke recurrence, stroke severity, morbidity, and mortality, earlier detection of occult cancer would allow for more rapid cancer treatment, which could improve patient outcomes (9). In this study, we aimed to assess the presence of cancer-related biomarkers in TIA patients with underlying cancer and to evaluate the long-term mortality as compared to TIA patients without active cancer.

## Methods

### Study cohort

Consecutive patients evaluated for TIA at our stroke center between 1 January 2015 and 31 December 2019 were retrospectively assessed for eligibility. All patients with a reliable diagnosis of TIA (as defined below) were included in this analysis. Patients with recurrent TIA after the index event were considered only once (index event). The present study adheres to the STROBE checklist for cohort studies, which was used to report the present study (see Supplementary material).

### Standard protocol approvals, registrations, and patient consents

The local ethics committee approved the study in accordance with Swiss law (Project ID: 2022-01560; Kantonale Ethikkommission Bern). According to the ethics committee's decision, no written consent was required from the patients for inclusion in this retrospective study. Study data are available upon reasonable request to the corresponding authors and after clearance by the local ethics committee.

### Definition of transient ischemic attack

In order to reliably identify patients with TIA, we classified patients according to the definition used in the Platelet-Oriented Inhibition in New TIA and Minor Ischemic Stroke Trial (POINT) as well as the National Institute of Neurological Disorders and Stroke (NINDS) criteria (10, 11). The final diagnosis of TIA was considered reliable when (1) neurological deficits were reversible within 24 h (time-based definition), (2) no signs of ischemia were present on acute brain imaging (in case of MRI: DWI [diffusion-weighted imaging] or perfusion deficit that might explain the focal neurological deficit leading to hospitalization; in case of CT: hypodensity in non-contrast CT or perfusion deficit; tissue-based definition), (3) clinical signs were consistent with a secure diagnosis of TIA (excluding isolated dizziness, double vision; sensory disturbances only affecting parts of a limb or the face).

### Definition of active cancer and occult cancer

Active cancer comprised the following two subgroups: known active cancer at the time of TIA or cancer diagnosed within 1 year after TIA and not known at the time of the initial TIA assessment (unknown cancer, named “occult cancer”). Known cancer was considered active when being newly diagnosed, recurrent, or treated within 6 months before the index TIA, or in case of metastatic spread (according to information obtained from our clinic information system) (12, 13). The limit of 12 months after TIA for the identification of occult cancer at the time of TIA was based on previous evidence (3, 14, 15). Occult cancer cases were either retrospectively identified in our clinic information system or documented in follow-up consultations. Focal non-melanoma skin cancers, such as basal cell carcinoma and squamous cell carcinoma, were not considered active cancers due to their tendency to remain local (16).

### Data collection

Five neurologists (MB, PC, JB, VZ, and PB) extracted the data analyzed in this study from the local emergency department information system (for out-patient treatment) and from the local stroke registry for hospitalized TIA patients (Swiss Stroke Registry). Baseline data included age at admission, gender, pre-stroke functional independence (defined as a modified Rankin scale [mRS] ≤2), cerebrovascular risk factors (such as hypertension, diabetes mellitus type II, hyperlipidemia, and history of smoking), National Institutes of Health Stroke Scale (NIHSS) on admission, presence of previous brain infarcts at baseline (covert brain infarction or old symptomatic brain infarction), baseline imaging modality (see Supplementary material—Imaging Analysis), and the following laboratory values at admission: albumin in g/L, CRP in mg/L, LDH in U/L, total cholesterol in mmol/L, low-density lipoprotein (LDL) cholesterol in mmol/L, triglycerides in mmol/L, D-dimer in μg/L, hemoglobin in g/L, leukocytes in g/L, and platelet count in g/L. The assigned TIA etiology at discharge was categorized according to the Trial of ORG 10172 in Acute Stroke Treatment (TOAST) classification and extracted from the clinical information system (17). TIA etiology was dichotomized as undetermined etiology and common etiologies according to the TOAST classification. The ABCD_2_ score was used to determine the risk of recurrent stroke after TIA (with a score of 6 or 7 points indicating a high-risk constellation). The occurrence of new cerebrovascular events (TIA or stroke) after discharge was evaluated through available follow-up reports. Deceased patients (long-term mortality rate) were identified through the Swiss Population Registry, which records the vital status of Swiss residents monthly. For surviving patients, the follow-up time was defined as the time from the index TIA to the last update of the Swiss Population Registry. For deceased patients, the follow-up time was defined as the time from the index TIA to the date of death.

### Statistical analysis

The characteristics of patients with and without active cancer are reported using median and interquartile range (IQR) for continuous variables and frequency (percentage) for categorical variables. Differences between both groups were assessed with Fisher's exact test for categorical variables and the Mann–Whitney U-test for continuous variables. Univariable and multivariable logistic regression models were used to evaluate for potential associations between TIA, active cancer, and the selected co-variables (male gender, age at admission, smoking history, CRP, D-dimer, LDH, platelet count, hemoglobin, leukocyte count, previous brain infarction on baseline imaging, and undetermined cause of TIA). Adjusted odds ratios (aORs) were reported with their corresponding 95% confidence intervals (95% CI). Long-term mortality rates for patients with and without cancer were reported via Kaplan–Meier curves using the log-rank test. Adjusted hazard ratios (aHRs) and their 95% CI were assessed with multivariable Cox regression analysis. Logarithmic transformation was applied to skewed distributed continuous variables. Continuous scales were inversed if lower laboratory values were associated with active cancer. In a secondary analysis comparing patients with occult malignancies vs. those without cancer, patients with previously known malignancies were excluded to avoid influencing the analysis of factors associated with malignancies (mainly blood parameter levels and the presence of multi-territory infarcts). No imputation was applied to compensate for missing data. Statistical analyses were performed with Stata 16 (StataCorp LLC).

## Results

### Study population

**Figure 1:**
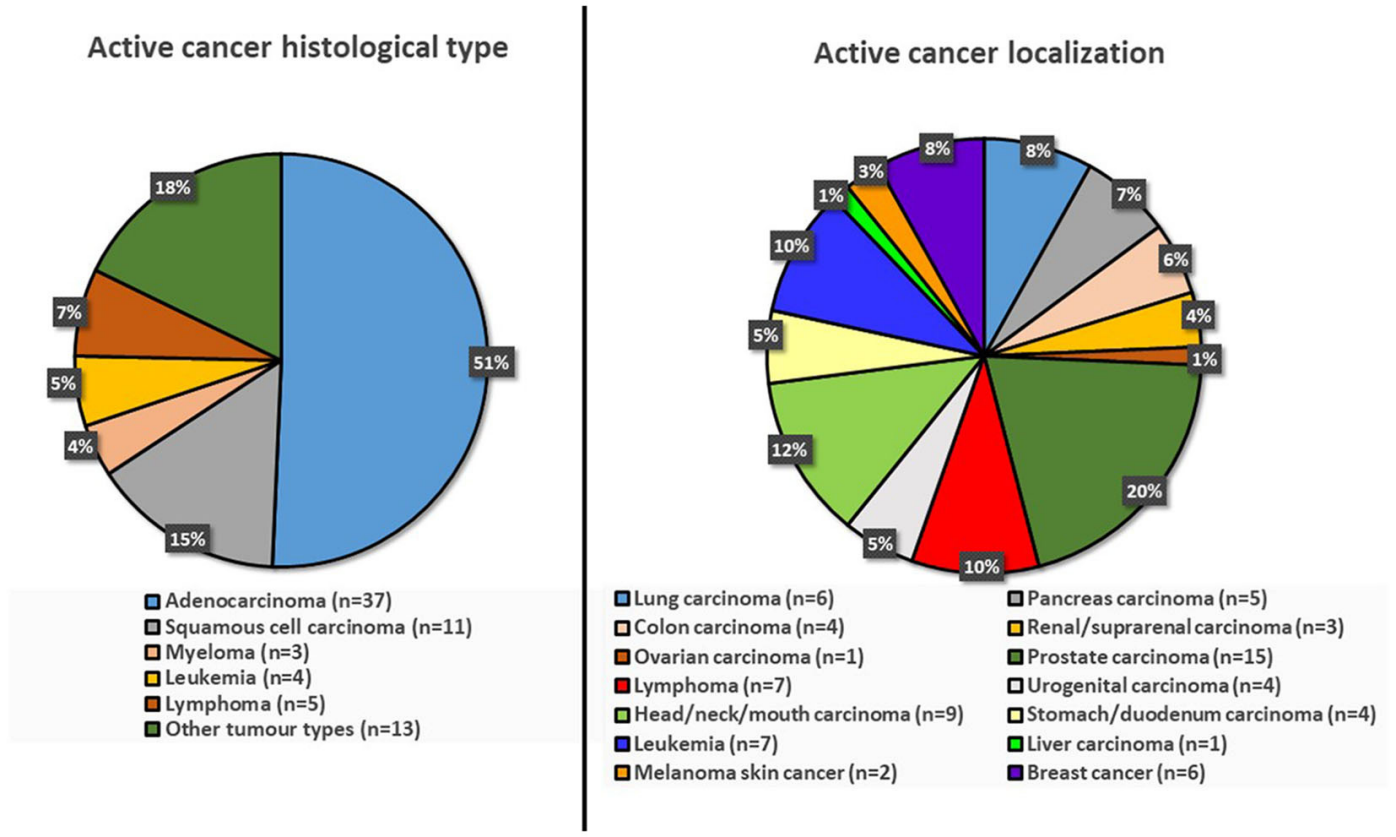
Histological types and locations of active cancer.

Between January 2015 and December 2019, 6,815 patients with a differential diagnosis of TIA were seen at our emergency department. Of those, 1,436 had a reliable diagnosis of TIA and were consequently included in this study (Figure 1—study flowchart). Active cancer was present in 72 patients (5%). Out of those, 55 patients (3.8%) presented with known active cancer and 17 patients (1.2%) suffered from occult cancer. The localization and histological type of active cancer patients are summarized in Figure 1. In patients with active known cancer at the time of TIA, the median time between cancer diagnosis and TIA was 408 days (IQR 143–1,476).

### Baseline characteristics

The baseline differences between patients with and without active cancer are summarized in Table 1. Patients with active cancer compared to patients without cancer were more often male (68% vs. 53%, *P* = 0.011), older (75.4 years vs. 71 years, *P* = 0.003), and more likely to smoke (38% vs. 20%, *P* = 0.001). TIA patients with active cancer presented with higher plasma levels of D-dimer, CRP, high-sensitive Troponin T, and INR at admission. They also had lower total cholesterol, LDL cholesterol, and hemoglobin. Groups did not differ regarding previous ischemic stroke lesions (as detected on cerebral imaging).

**Table 1:** Comparison of baseline characteristics between patients with active cancer vs. no cancer.

	**All patients (*N* = 1,436)**	**No cancer (*N* = 1,364)**	**Active cancer (*N* = 72)**	***P*-value**
**Baseline**				
Age at admission (median, IQR)	71 (59–80.4)	71 (59–82.0)	75.4 (70–82.4)	0.003
Gender (male) No./total No. (%)	767/1,436 (53.4%)	718/1,364 (52.6%)	49/72 (68.0%)	0.011
Prior independence (mRS ≤ 2) No./total no. (%)	855/954 (89.6%)	821/912 (90%)	34/42 (81%)	0.069
**Risk factors No/total No. (%)**				
Diabetes	204/1,399 (14.6%)	190/1,330 (19.0%)	14/69 (20.3%)	0.16
Hypertension	787/1,399 (56.3%)	745/1,330 (56.0%)	42/69 (60.9%)	0.46
Atrial fibrillation	158/1,398 (11.3%)	147/1,326 (11.1%)	11/72 (15.9%)	0.24
Dyslipidemia	687/1,398 (49.1%)	652/1,329 (49.1%)	35/69 (50.7%)	0.81
Smoking	292/1,396 (20.9%)	266/1,061 (25%)	26/69 (37.7%)	0.02
History of stroke	198/1,402 (14.1%)	158/1,330 (11.9%)	16/70 (17.1%)	0.05
History of TIA	170/1,400 (12.1%)	18/83 (21.7%)	9/43 (21.0%)	0.19
Previous brain infarctions	308/1,422 (21.6%)	293/1,351 (21.7%)	15/71 (21.1%)	1.00
Venous thromboembolism	12/1,436 (0.8%)	8/1,364 (0.6%)	4/72 (5.6%)	0.002
**TIA characteristics**				
NIHSS on admission (median, IQR)	0 (0–1)	0 (0–1)	0 (0–2)	0.18
MRI as baseline imaging No./total No. (%)	1,117/1,332 (83.9%)	1,061/1,267 (83.7%)	56/65 (86.2%)	0.73
ABCD_2_ score (median, IQR)	4 (3–5)	4 (3–5)	4 (3–5)	0.88
ABCD_2_ score ≥6 (high risk)	195/1,436 (13.6%)	183/1,364 (13.4%)	12/72 (16.7%)	0.62
**TIA etiology (TOAST)**				
**No./total No. (%):**				
Cardioembolic	125/1,394 (9%)	118/1,325 (8.9%)	7/69 (10.1%)	0.35
Small-vessel occlusion	21/1,394 (1.5%)	20/1,325 (1.5%)	1/69 (1.4%)	
Competing etiologies	47/1,394 (3.4%)	43/1,325 (3.2%)	4/69 (5.8%)	
Large-artery atherosclerosis	97/1,394 (7%)	89/1,325 (6.7%)	8/69 (11.6%)	
TIA of other determined etiology	22/1,394 (1.6%)	21/1,325 (1.6%)	1/69 (1.4%)	
TIA of undetermined etiology	1,082/1,394 (77.6%)	1,034/1,325 (78%)	48/69 (69.6%)	
**Baseline laboratory findings**				
Albumin in g/L (median, IQR)	34 (31–37)	34 (31–37)	33.5 (29.5–37.5)	0.60
C-reactive protein in mg/L (median, IQR)	2 (1–5)	2 (1–4)	3.5 (1.5–9)	< 0.001
LDH in U/L (median, IQR)	385 (337–440)	384.5 (337.5–437)	433 (336.5–508.5)	0.01
Troponin T-high-sensitive in ng/L (median, IQR)	8.36 (4.85–16.19)	8.1 (4.82–16)	11.9 (5.76–22.1)	0.006
Total cholesterol in mmol/L (median, IQR)	4.82 (3.96–5.65)	4.84 (4.01–5.67)	4.42 (3.36–4.95)	< 0.001
LDL cholesterol in mmol/L (median, IQR)	2.60 (1.92–3.30)	2.62 (1.92–3.33)	2.37 (1.88–2.91)	0.025
Leukocyte count in G/L (median, IQR)	7.46 (6.2–9.1)	7.5 (6.2–9.13)	7.15 (5.78–8.40)	0.15
Hemoglobin in g/L (median, IQR)	138 (128–147)	138 (128–147)	127 (113–145)	< 0.001
Platelet count in G/L (median, IQR)	228 (194–270)	228 (194–270)	209.5 (174–279.5)	0.19
Fibrinogen in g/L (median, IQR)	2.9 (2.46–3.45)	2.9 (2.46–3.43)	3 (2.48–3.6)	0.42
INR (median, IQR)	1 (0.96–1.05)	1 (0.96–1.05)	1.02 (0.97–1.11)	0.007
D-dimer in μg/L (median, IQR)	444 (247–819)	434 (244–782)	864.5 (364–2577.5)	< 0.001
HbA1c in % (median, IQR)	5.6 (5.4–6)	5.6 (5.4–6)	5.6 (5.3–6)	0.62
**Outcome**				
Long-term follow-up time in days (median, IQR)	1,699 (1,212–2,251)	1,732 (1,247–2,261)	807 (410–1,577)	< 0.001
Recurrent stroke	74/1,250 (5.9%)	69/1,184 (5.8%)	5/66 (7.6%)	0.59
Recurrent TIA	88/1,248 (7%)	82/1,182 (6.9%)	6/66 (9.1%)	0.46
Long-term deaths No./total No. (%)	292/1,096 (26.6%)	252/1,039 (24.3%)	40/57 (70.2%)	< 0.001

IQR indicates interquartile range; LDH, lactate dehydrogenase; LDL, low-density lipoprotein; MRI, magnetic resonance imaging; NIHSS, National Institutes of Health Stroke Scale; TIA, transient ischemic attack.

The denominator differs according to the overall availability of the data (on the respective risk factor or laboratory finding).

### Characteristics of cancer-related TIA

**Figure 2:**
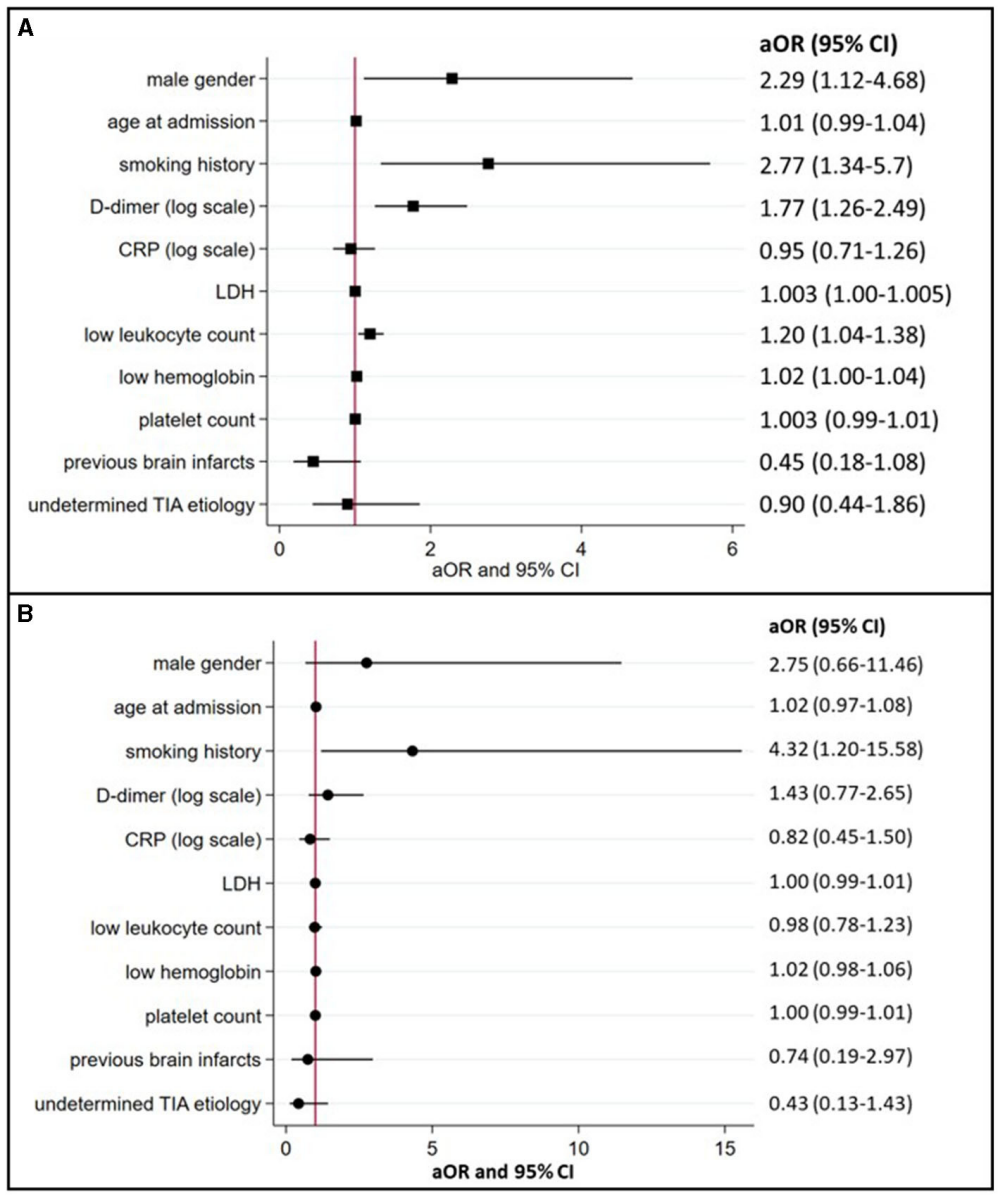
Associations between TIA patients with active or occult cancer and preselected demographics, risk factors, and biomarkers (multivariate logistic regression model). **(A)** (active cancer) and **(B)** (occult cancer) summarize the association between cancer in TIA patients and preselected demographics, risk factors, and blood biomarkers with corresponding aOR and 95% confidence intervals. aOR, indicates adjusted odds ratios; CI, confidence intervals; CRP, C-reactive protein; LDH, lactate dehydrogenase; and TIA, transient ischemic attack.

In multivariable logistic regression analyses (summarized in Figure 2A), cancer-related TIA (active cancer overall) was associated with male sex (aOR 2.29, 95% CI 1.12–4.68) and a history of smoking (aOR 2.77, 95% CI 1.34–5.7). The presence of active cancer was also associated with higher D-dimer (aOR 1.77, 95% CI 1.26–2.49), LDH (aOR 1.003, 95% CI 1.00–1.005), lower leukocyte count (aOR 1.20, 95% CI 1.04–1.38), and lower hemoglobin (aOR 1.02, 95% CI 1.00–1.04).

In 15 of the 17 occult cancer patients (88%), diagnosis was made after discharge. The median time delay to diagnosis was 180 days (IQR 116–226). In multivariable logistic regression analyses after excluding patients with known active cancer (summarized in Figure 2B), occult cancer at the time of TIA was only associated with a history of smoking (aOR 4.32, 95% CI 1.20–15.58).

### Long-term outcomes in cancer-related TIA

Long-term follow-up data as obtained from the Swiss Population Registry were available for 1,021 (74.9%) patients without cancer as compared to 57 of all active cancer patients (79.1%) and 12 (100%) in the subgroup of occult cancer patients. The median long-term follow-up time for patients with active cancer was 807 days (IQR 410–1,577) and for patients without active cancer 1,732 days (IQR 1,247–2,261).

**Figure 3:**
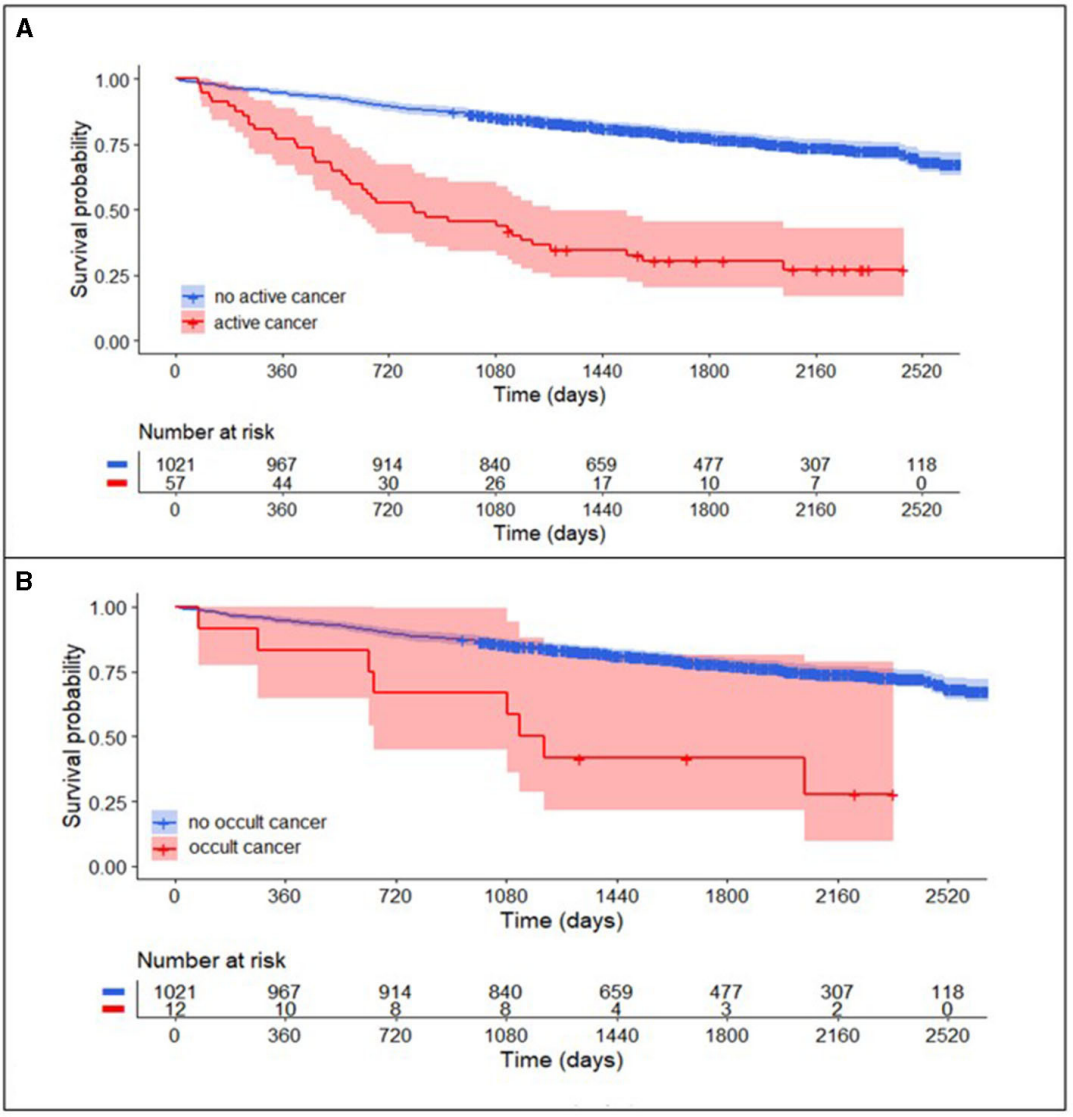
Long-term survival curve in TIA-patients with and without cancer. **(A)** Patients with active cancer (combined known active cancer and occult cancer; red) compared to patients without active cancer (blue) had higher mortality rates in the long-term follow-up after TIA (log-rank test, *P* < 0.001). After the exclusion of patients with known active cancer **(B)**, mortality rates in patients with occult cancer (red) compared to patients without active cancer (blue) in the long-term follow-up remained higher (log-rank test, *P* < 0.001).

**Figure 4:**
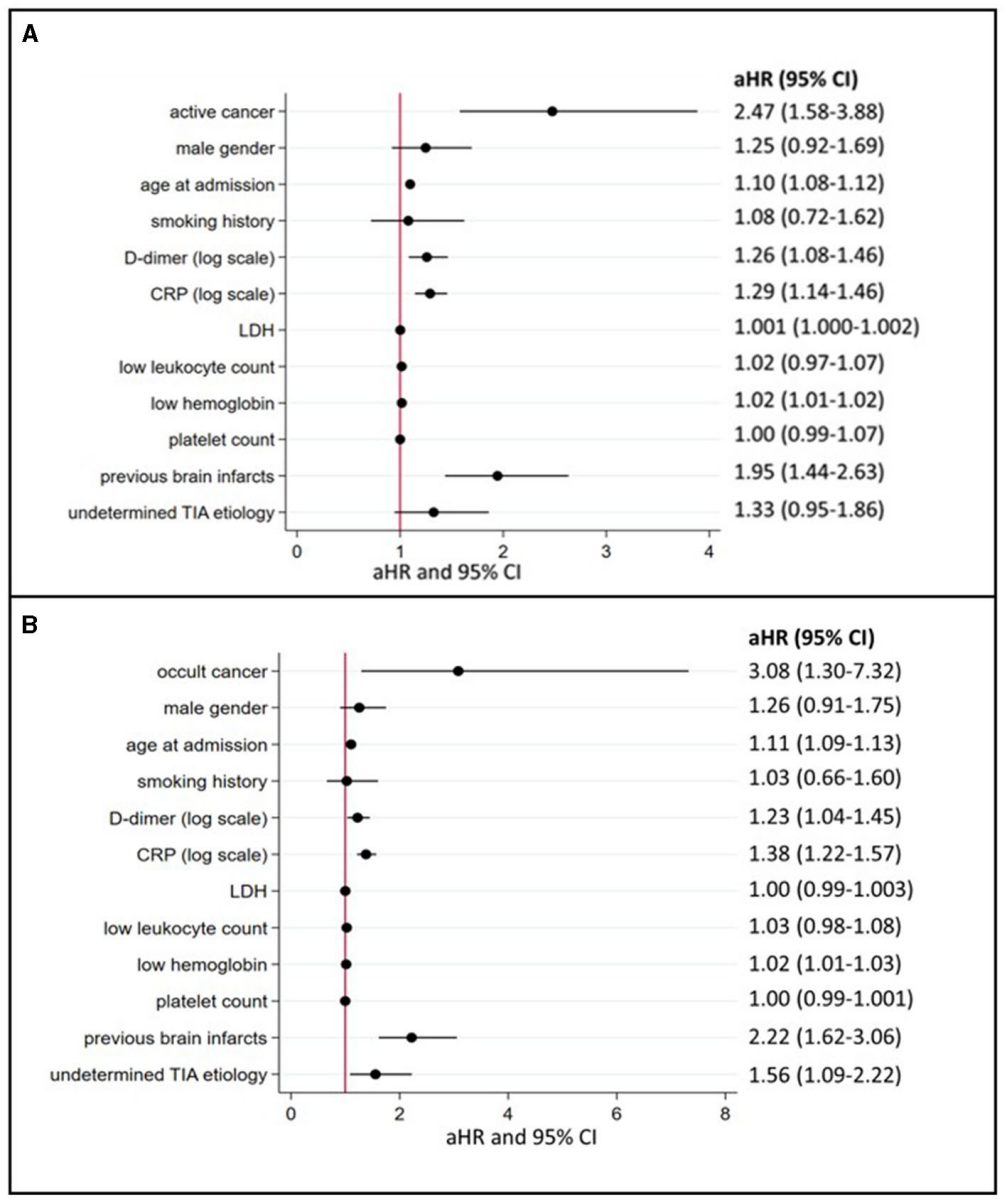
Predictors of long-term mortality in TIA patients with active or occult cancer (multivariate logistic regression model). **(A)** depicts the aHR from the multivariate Cox regression analyses regarding long-term mortality depending on the presence of active cancer and other cancer-related factors (for occult cancer, see **(B)**. aHR indicates adjusted hazard ratios; CI, confidence intervals; CRP, C-reactive protein; LDH, lactate dehydrogenase and TIA, transient ischemic attack.

Patients with active cancer had a higher mortality rate during long-term follow-up (Figure 3A, log-rank test, *P* < 0.001) as compared to patients without cancer. In multivariable Cox regression analyses (Figure 4A), long-term mortality was strongly associated with active cancer (aHR 2.47, 95% CI 1.58–3.88) and previous brain infarcts (aHR 1.95, 95% CI 1.44–2.63). A weaker association with long-term mortality was also found for higher D-dimer and CRP and lower hemoglobin (Figure 4A).

In the subgroup of patients with occult cancer, the long-term mortality rate remained significantly higher than in patients without cancer (Figure 3B, log-rank test, *P* < 0.001). The median long-term follow-up time for patients with occult cancer was 1,163 days (IQR 640–1,861) and for patients without occult cancer 1,732 days (IQR 1,247–2,261). In multivariable Cox regression analyses (Figure 4B), long-term mortality was strongly associated with occult cancer (aHR 3.08, 95% CI 1.30–7.32), previous brain infarcts (aHR 2.22, 95% CI 1.62–3.06), and undetermined TIA etiology (aHR 1.56, 95% CI 1.09–2.22). Weaker significant associations were found with age at admission, elevated D-dimer, higher CRP, and lower hemoglobin.

## Discussion

The main findings of this study are TIA patients with active cancer present with characteristics and biomarkers similar to cancer-related stroke and experience higher mortality rates as compared to controls. As for occult cancer, the majority of cases were diagnosed after discharge and not during hospitalization.

Elevated D-dimer, a fibrin degradation product suggesting blood clot predilection or formation, is the most frequent biomarker associated with cancer in stroke patients (18, 19). Rosenberg et al. (20) retrospectively demonstrated that stroke patients with elevated D-dimer levels (median 2,520 μg/L, IQR 1,250–4,080) had a higher risk of being diagnosed with cancer when undergoing a whole-body CT in the post-stroke phase. In our study, an elevated D-dimer at admission was strongly associated with active cancer. This observation suggests a similar paraneoplastic coagulopathy phenomenon in cancer-related TIA compared to patients with cancer-related stroke. In our cohort, CRP levels were higher in the cancer group. However, there was no association with cancer-related TIA in the multiple logistic regression analyses. Karlinska et al. documented a median CRP level of 21 mg/L (IQR 4–76) in stroke patients with cancer, which is considerably higher than the median CRP level of 3.5 mg/L (IQR 1.5–9) found in our study. A potential explanation might be that the paraneoplastic inflammatory process is less active in patients with TIA (and therefore not yet leading to a subsequent permanent ischemia) (21) Cancer-related TIA was associated with decreased leukocyte count. Since there is currently no evidence of leukopenia or leukocytosis in cancer-related stroke, one possible explanation is the effect of cancer treatments (especially radiotherapy) known to cause lymphopenia (19, 22, 23). Reduced hemoglobin and elevated LDH, both correlating with cancer-related stroke, were weakly associated with cancer-related TIA (19, 24). A similar association was found for an elevated hs-Troponin T in our cohort. While being non-specific due to a number of potential causes, higher hs-Troponin T levels could be detected recently in ischemic stroke patients with concomitant active cancer (25).

Although not reaching statistical significance, there was an inverse correlation between cancer-related TIA and previous brain infarction on MRI. This finding was observed in active cancer patients. Currently, there is no persuasive explanation. It may be assumed that during the initial stroke work-up, a potential (paraneoplastic) coagulation disorder might have been detected and consecutively treated (e.g., anticoagulation). There was no difference between groups when looking at the ABCD_2_ score which predicts recurrent cerebrovascular events after TIA (even for high-risk patients with an ABCD_2_ score of six or higher, Table 1). One explanation might be that cancer and/or paraneoplastic coagulation disorder are not part of the score. Overall, active cancer is known to be an independent risk factor for recurrent events (9).

Cancer-related stroke is known to be associated with multiterritory infarcts (26). Finelli et al. described the “three-territory sign“ as DWI restriction in all three vascular territories (bilateral anterior and posterior circulation) (26). The “three-territory sign” is highly suggestive of cancer-related stroke in the absence of an identifiable cause (e.g., atrial fibrillation) (27). To identify a potential radiological biomarker in cancer-related TIA, we assessed the presence of previous brain infarcts at baseline (covert brain infarction or old symptomatic brain infarction). No association between previous brain infarcts and either active cancer or occult cancer patients was found. In summary, features of paraneoplastic coagulopathy appear to be present in TIA patients with active cancer. Similar to ischemic stroke, the identification of a paraneoplastic coagulopathy seems to be of importance and has to be taken into consideration (especially in cases of an unknown etiology). These similar clinical characteristics could help guide secondary prevention management in these patients, including possible trials of anticoagulation.

Regarding the determination of predictors for a new cancer diagnosis within 1 year after TIA, this study did not identify potential predictors of occult malignancy except smoking habits. D-dimer levels were the same in TIA patients with occult cancer compared to patients without cancer. Further studies are needed to evaluate whether available predictive scores for occult cancer-related stroke can be used in TIA patients. Despite the low sensitivity of the above-mentioned biomarkers in occult malignancy, mortality rates in TIA patients with occult and active malignancy did not differ in our cohort (and were higher compared to patients without cancer). The abovementioned biomarkers might therefore not be suitable candidates for initial screening and assessment. However, especially in cases of unknown TIA etiology (and a history of smoking), our results support a thorough diagnostic work-up including the search for occult cancer and subsequent paraneoplastic coagulopathies. TIA, as the first manifestation of occult cancer, could accelerate the cancer diagnosis and thus potentially improve the prognosis of these patients.

## Limitations

Our study has several limitations. First, due to the retrospective study design, all attributed biases apply. The true incidence of occult cancer and recurrent TIA/stroke might be underestimated due to diagnoses made at other centers during follow-up. Second, because of the single-center design, the findings may not be generalizable. Third, the stage of the disease (cancer) at the time of TIA onset was not documented. This limits conclusions regarding the effect of advanced disease on paraneoplastic activity. Fourth, due to the small number of TIA attributable to occult cancer, effects or associations might be imprecise. In addition, indirect detection of a potential pro-thrombotic state (e.g., microembolic signaling in transcranial color Doppler) was not available. Moreover, it has to be mentioned that the complete etiological assessment of many patients was completed in an outpatient setting. This could lead to a misinterpretation of some of the baseline information (e.g., TOAST criteria) as the final etiology might not go along with the one that was initially suspected.

## Conclusion

Our study indicates that cancer-related TIA is not uncommon and blood biomarkers known to be associated with cancer-related stroke are also present in TIA patients with active cancer. Similarly, patients with cancer-related TIA experience higher mortality rates. These biomarkers are smoking history, elevated D-dimer, elevated LDH, and low hemoglobin. Identification and analysis of these biomarkers might help to facilitate diagnosis and treatment which might be of clinical relevance. Future studies should validate these findings in prospective multicenter cohorts and investigate the optimal treatments for cancer-related TIA.

## Data availability statement

The datasets presented in this article are not readily available because of ethical and privacy restrictions. Requests to access the datasets should be directed to the corresponding authors.

## Ethics statement

The study involving humans was approved by the Kantonale Ethikkommission Bern (Project ID: 2022-01560). The study was conducted in accordance with the local legislation and institutional requirements. Written informed consent for participation was not required from the participants or the participants' legal guardians/next of kin in accordance with the national legislation and institutional requirements.

## Author contributions

MB: Conceptualization, Data curation, Formal analysis, Investigation, Writing—original draft. PC: Data curation, Writing—review and editing. JB: Data curation, Writing—review and editing. VZ: Data curation, Writing—review and editing. MK: Writing—review and editing. MM: Writing—review and editing. SB-G: Writing—review and editing. AM: Writing—review and editing. TM: Writing—review and editing. TH: Conceptualization, Writing—review and editing. UF: Writing—review and editing. JK: Writing—review and editing. MH: Writing—review and editing. DS: Writing—review and editing. MA: Writing—review and editing. TP: Writing—review and editing. MDB: Writing—review and editing. BN: Writing—review and editing. SJ: Conceptualization, Resources, Supervision, Writing—review and editing. PB: Conceptualization, Data curation, Project administration, Supervision, Writing—original draft, Writing—review and editing.
