# Supplementary material for: Transient ischemic attacks in patients with active and occult cancer
Source: Front Neurol. 2023 Sep 28;14:1268131. doi: 10.3389/fneur.2023.1268131 (PMC10568457; doi:10.3389/fneur.2023.1268131)
Supplement: Supplementary file 1 [file Data_Sheet_1.docx]

**SUPPLEMENTAL MATERIAL**

**Transient ischemic attacks in patients with active cancer**

Morin Beyeler MD^1,2^, Pasquale Castigliego MS^1^, Joel Baumann MS^1^, Victor Ziegler MS^1^, Moritz Kielkopf MD^1^, Madlaine Mueller MD^1^, Stefan Bauer MD^1^, Adnan Mujanovic MD^3^, Thomas Raphael Meinel MD^1^, Thomas Horvath MD^1^, Urs Fischer MD^1,4^, Johannes Kaesmacher MD^3^, Mirjam Heldner MD^1^, David Seiffge MD^1^, Marcel Arnold MD^1^, Thomas Pabst MD^5^, Martin D. Berger MD^5^, Babak B. Navi MD^6^, Simon Jung MD^1^*, Philipp Bücke MD^1^*

* equal contribution

1) Department of Neurology, Inselspital, Bern University Hospital, and University of Bern, Switzerland

2) Graduate School for Health Sciences, University of Bern, Switzerland

3) Institute for Diagnostic and Interventional Neuroradiology, Inselspital, Bern University Hospital, and University of Bern, Switzerland

4) Neurology Department, University Hospital of Basel, University of Basel, Basel, Switzerland

5) Department of Medical Oncology, Inselspital, Bern University Hospital, and University of Bern, Switzerland

6) Clinical and Translational Neuroscience Unit, Feil Family Brain and Mind Research Institute and Department of Neurology, Weill Cornell Medicine, New York, New York, USA

**Supplementary Method**

**Imaging Analysis**

Brain MRI was performed on a 1.5T or 3T MR imaging scanner (1.5T: Magnetom Avanto or Magnetom Aera; 3T: Magnetom Verio; Siemens). CT of the brain was performed on a 128-row CT scanner (Siemens SOMATOM Edge, Siemens Erlangen, Germany) by use of CarekV (Quality reference of 120 kV), modulated milliampere-seconds (mAs) with CareDose4D (Quality reference of 290 mAs), with 1.0mm section thickness. MRI perfusion maps were generated using the Olea Sphere Software environment (Olea Sphere v2.3; Olea Medical, La Ciotat, France). CT perfusion images were processed by the post-processing software syngo.via (Siemens). The following perfusion maps and thresholds were considered: Relative cerebral blood volume (rCBV), relative cerebral blood flow (rCBF) and time to maximum (Tmax). Presence of an ischemic core (defined as a reduced rCBV <2 mL/100 g and rCBF <25 mL/100 g/min) or with tissue at risk (defined as a Tmax >6 sec) in the territory of the suspected TIA were assessed using available radiological reports.^1–3^

1. Asdaghi N, Coutts SB. The role of urgent imaging in the diagnosis and management of patients with TIA and minor stroke. *Imaging Med*. 2013;5(1):25-33. doi:10.2217/iim.13.3

2. Goyal M, Ospel JM, Menon B, et al. Challenging the Ischemic Core Concept in Acute Ischemic Stroke Imaging. *Stroke*. 2020;(October):3147-3155. doi:10.1161/STROKEAHA.120.030620

3. Lui YW, Tang ER, Allmendinger AM, Spektor V. Evaluation of CT perfusion in the setting of cerebral ischemia: Patterns and pitfalls. *Am J Neuroradiol*. 2010;31(9):1552-1563. doi:10.3174/ajnr.A2026

**Supplementary Figures**

**
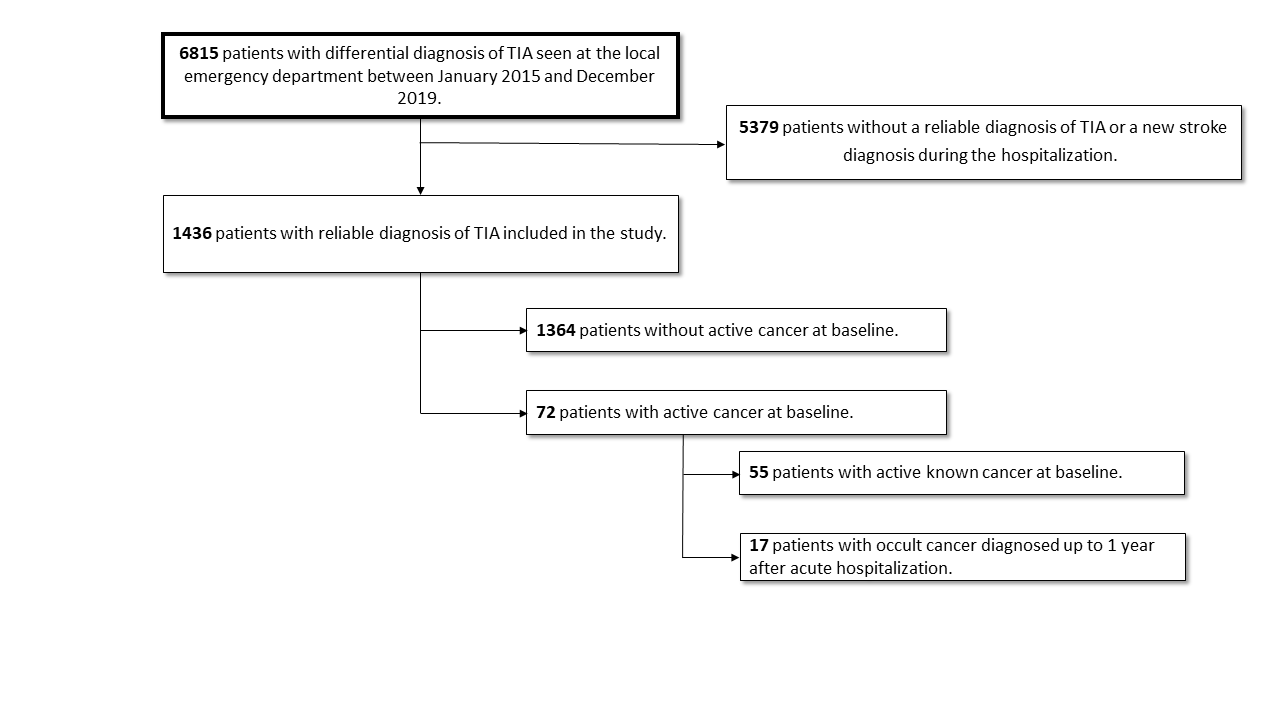
**

**eFigure I** – Study flowchart.
Inclusion and exclusion of study participants. TIA indicates transient ischemic attack.
